# Tracking the Prevalence of Antibiotic Resistance in *Enterococcus* Within the Spanish Poultry Industry: Insights from a One Health Approach

**DOI:** 10.3390/antibiotics14010016

**Published:** 2024-12-30

**Authors:** Josep Garcia-Llorens, Isaac Monroy, Jan Torres-Boncompte, Jose M. Soriano, Pablo Catalá-Gregori, Sandra Sevilla-Navarro

**Affiliations:** 1 Centro de Calidad Avícola y Alimentación Animal de la Comunidad Valenciana (CECAV), 12539 Alquerias del Niño Perdido, Spain; j.garcia@cecav.es (J.G.-L.); i.monroy@cecav.org (I.M.); j.torres@cecav.es (J.T.-B.); p.catala@cecav.org (P.C.-G.); 2 Food & Health Lab, Institute of Materials Science, University of Valencia, 46980 Valencia, Spain; jose.soriano@uv.es; 3 Joint Research Unit on Endocrinology, Nutrition and Clinical Dietetics, University of Valencia-Health Research Institute La Fe, 46026 Valencia, Spain

**Keywords:** *Enterococcus*, antimicrobial resistance, surveillance, one health, poultry

## Abstract

**Background**/**Objectives**: Antimicrobial resistance (AMR) in *Enterococcus* species from poultry production represents a significant public health threat due to the potential transmission of AMR through the food chain. This study aimed to examine the relative prevalence, resistance patterns, and mannitol fermentation capacity of *Enterococcus* isolates from various poultry production systems in Spain over a seven-year period (2017–2023). **Methods**: A total of 215 *Enterococcus* isolates were analyzed. Phenotypic assessments were conducted to determine resistance rates and metabolic capacities, while genotypic characterization focused on detecting vancomycin-resistance genes (*vanA*, *vanB*, *vanC*, and *vanD*). **Results**: *Enterococcus faecalis* (62.3%) and *Enterococcus faecium* (29.77%) were the predominant species, primarily isolated from broilers (74.88%), with the highest frequency observed in one-week-old chicks (31.16%). High resistance rates to tetracyclines and streptogramins were identified, while resistance to vancomycin (0.47%) and tigecycline (3.03%) was low. Interestingly, a significant reduction in tetracyclines resistance was shown in this period for *Enterococcus faecalis* (from 100% to 70% (2017–2023) and *Enterococcus faecium* (from 100% to 40% (2018–2023)). Multidrug resistance (MDR) was detected in 26.98% of isolates. Mannitol fermentation tests revealed high metabolic capacity in *Enterococcus faecalis* (99.25%) and *Enterococcus faecium* (96.88%), associated with adaptability and virulence potential. Genotypic analysis showed a very low prevalence of *vanB* and *vanC* genes. **Conclusions**: These findings highlight the critical need for targeted surveillance and intervention strategies in poultry production to mitigate the risks posed by MDR *Enterococcus* to public health.

## 1. Introduction

*Enterococcus* is a versatile genus of Gram-positive bacteria commonly found in various environments, including the gastrointestinal tracts of humans and animals [1]. As a commensal bacterium, it is typically present in the gut of poultry, contributing positively to their growth and feed efficiency [2,3]. However, disruptions in the intestinal microbiota can lead to invasive infections by *Enterococcus* [4,5], making it an emerging pathogen of clinical importance [6].

Currently, more than 60 *Enterococcus* species have been described based on data from the List of Prokaryotic Names [7]. Nevertheless, some species, such as *Enterococcus faecium* (*E. faecium*) and *Enterococcus faecalis* (*E. faecalis*), among others, are closely associated with clinically significant issues in the poultry sector [8,9,10]. These species are notable for their ability to develop antibiotic resistance, survival in harsh environments, and adaptability to different hosts, posing risks to both poultry health and the human food chain [11,12,13].

In poultry, *E. faecium* and *E. faecalis* are implicated in most *Enterococcus*-related infections [14], particularly in the deaths of one-day-old chicks [15]. In adult poultry, these pathogens can cause bacteremia, pulmonary hypertension, amyloid arthropathy, encephalomalacia, and other neurological disorders [14]. Primary routes of transmission include the fecal contamination of eggs, as well as aerosol or oral transmission [16]. While *E. faecalis* is a ubiquitous pathogen across productive species, *E. faecium* is less prevalent but more frequently antibiotic-resistant [17]. Additionally, other species, such as *Enterococcus hirae* (*E. hirae*), *Enterococcus durans* (*E. durans*), and *Enterococcus casseliflavus* (*E. casseliflavus*) [18,19,20] have been identified in poultry. All these species can cause various diseases, including enteritis and septicemia, which are significant concerns for animal health, economic impact, and the transmission of antibiotic resistance [21,22,23].

Antimicrobial resistance (AMR) is a global public health concern exacerbated by the use of antimicrobials in animal and human medicine [24]. *Enterococcus* is clinically significant due to its intrinsic resistance to various antimicrobials, like β-lactams, and its ability to acquire resistance genes via horizontal gene transfer, leading to resistance against a broad range of antibiotics [1,25]. Thus, *Enterococcus* is part of the ESKAPE pathogens group, comprising *Enterococcus faecium*, *Staphylococcus aureus*, *Klebsiella pneumoniae*, *Acinetobacter baumannii*, *Pseudomonas aeruginosa*, and *Enterobacter* spp., i.e., pathogens noted by the World Health Organization (WHO) as microorganisms for which the development of new antimicrobials treatment is considered urgent [26,27,28]. In the poultry industry, multidrug-resistant (MDR) *Enterococcus* strains have become a serious issue. Despite restrictions on antibiotic use, the prevalence of MDR *E.faecalis* remains high. Poultry has been identified as a reservoir for resistant enterococci, which act as vectors for the spread AMR. In fact, the latest data reported by the European Food Safety Authority (EFSA) [29] showed *E. faecalis* to be among the most relevant AMR bacteria in the European Union in poultry. This is due to its increasing clinical importance in the last decades, challenges associated with its treatment (often due to a late etiological diagnosis) and its wide distribution, together with its high levels of resistance found for certain antimicrobials, which are also widely used for its treatment (lincosamides and spectinomycin). *Enterococcus* is a clear example of a pathogen to be controlled under a One Health approach. In fact, several investigations evidence a connection between animal and human antibiotic use, with critical AMR genes shared across pathogens. Notably, hospital-adapted *E. faecium* strains originated from animal-associated strains, reinforcing the link between animals and human health [30]. Thus, there is a need for collaboration across sectors and disciplines to address health challenges, such as AMR.

Some species of *Enterococcus*, such as *E. faecalis*, are intrinsically resistant to different first-line antimicrobial agents (e.g., low-level resistance to β-lactams and aminoglycosides) and have the capacity to acquire resistance to several other antimicrobial agents, including last-resort antibiotics, such as glycopeptides [8,31]. Vancomycin-resistant enterococci (VRE) are of particular concern, since the glycopeptide vancomycin is being used as a last resort drug for the treatment of infections caused by MDR *Enterococcus* and *Staphylococcus* [32]. Furthermore, it has been suggested that human colonization by VRE could occur through the food chain [31,33,34], given the presence of antimicrobial resistance genes (*vanA*) in strains found in animals [35,36].

In addition, given that several studies developed worldwide have shown that *Enterococcus* is an emerging pathogen in the poultry sector but information about it in Spain remains limited, this study aims to address this gap by determining the relative prevalence of *Enterococcus* species in different poultry production systems, assessing their mannitol fermentation capacity, and analyzing their antibiotic resistance patterns. Isolates collected from poultry farms over a seven-year period will provide insights into the persistence and dynamics of these organisms in poultry environments.

## 2. Results

### 2.1. Relative Prevalence of Clinical Enterococcus Isolates from 2017 to 2023

The matrix-assisted laser desorption/ionization time-of-flight (MALDI-TOF) analysis of 215 *Enterococcus* isolates confirmed that *E. faecalis* (n = 134/215; 62.33%) and *E. faecium* (n = 64/215; 29.77%) were the predominant species. Other species included *E. hirae* (n = 10/215; 4.65%), *E. durans* (n = 4/215; 1.86%), *Enterococcus gallinarum* (*E. gallinarum*) (n = 2/215; 0.93%), and *E. casseliflavus* (n = 1/215; 0.47%) (Table 1).

**Figure 1:**
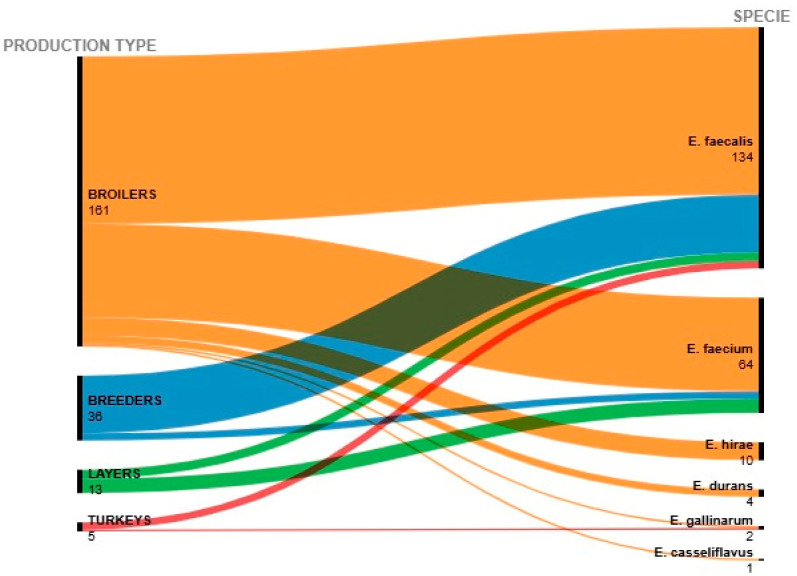
Alluvial diagram representing the distribution of *Enterococcus* species across different poultry production types (broilers, turkeys, breeders, and layers) from 2017 to 2023. The prevalence of each species is described on the right, and the production type is described on the left part. (Diagram created using RawGraph v.2) [37].

The distribution patterns of *Enterococcus* species across different poultry production types—broilers, turkeys, breeders, and layers—are represented with an alluvial diagram (Figure 1). The diagram highlights the strong dominance of *E. faecium* and *E. faecalis*, particularly in broilers and breeders. The flow patterns suggest that while *E. faecium* and *E. faecalis* were consistently detected, other species, such as *E. durans*, *E. casseliflavus*, *E. gallinarum*, and *E. hirae*, appeared more sporadically and seemed less dependent on production type.

### 2.2. Mannitol Fermentation Assessment

Mannitol fermentation capability was evaluated as an indicator of metabolic versatility among *Enterococcus* species (Table 2). Among the strains tested, *E. faecalis* demonstrated the highest fermentation ability, with 99.25% (133/134) of isolates showing positive results (++). Similarly, *E. faecium* exhibited substantial fermentation capability, with 96.88% (62/64) of isolates producing positive results (++), while 1.56% showed medium fermentation (+) and 1.56% yielded negative outcomes (-). These findings underscore the adaptability of *E. faecalis* and *E. faecium*, potentially correlated with their prevalence and possible virulence in poultry environments. In contrast, *E. hirae* and *E. durans* exhibited limited fermentation ability, with no positive results recorded for either species (-). However, medium fermentation capacity (+) was observed in 40% of *E. hirae* isolates and 50% of *E. durans* isolates, indicating their metabolic activity, which may be relevant in specific ecological niches. Interestingly, both *E. gallinarum* and *E. casseliflavus* achieved 100% positive results (++), although their low representation in the dataset (n = 2 and n = 1, respectively) warrants cautious interpretation. These species, while sporadic in occurrence, demonstrate complete fermentation capacity, suggesting a potential for metabolic specialization. The variability in mannitol fermentation among *Enterococcus* species highlights the differing metabolic strategies and possible ecological roles of these bacteria. *E. faecalis* and *E. faecium* dominate in both metabolic adaptability and prevalence, reinforcing their significance in poultry-associated environments.

### 2.3. Antimicrobial Susceptibility Test

Concerning antimicrobial resistance, 26.98% (58/215) of the *Enterococcus* isolates were MDR. Statistically significant differences were observed between the antibiotic resistance profiles. The resistance rates were as follows: tetracycline (TET, 70.70%), erythromycin (ERY, 38.60%), ciprofloxacin (CIP, 21.40%), daptomycin (DAP, 10.23%), chloramphenicol (CHL, 7.44%), gentamicin (GEN, 4.65%), vancomycin (VAN, 0.47%), and ampicillin (AMP, 0.47%), while no resistance was observed to teicoplanin (TEI, 0%) or linezolid (LZD, 0%). Among antimicrobials with breakpoints established exclusively for *E. faecium*, 87.50% (56/64) of isolates were resistant to quinupristin–dalfopristin (SYN). Additionally, 3.03% (6/198) of isolates, including both *E. faecium* and *E. faecalis*, exhibited resistance to tigecycline (TGC).

A total of 37 different patterns were identified, with 24 replicated in 2 or more strains. The most predominant phenotypic resistance pattern was TET (n = 50; 23.26%) and TET-ERY (n = 35; 16.28%) followed by the TET-CIP-ERY pattern (n =9; 4.19%). Resistance profiles involving six antibiotics were also observed, although less frequently. For instance, the SYN-TET-DAP-CIP-ERY-CHL pattern was identified in only two isolates (0.93%), while other six-antibiotic resistance patterns were even rarer, each accounting for just 0.47% of isolates.

**Figure 2:**
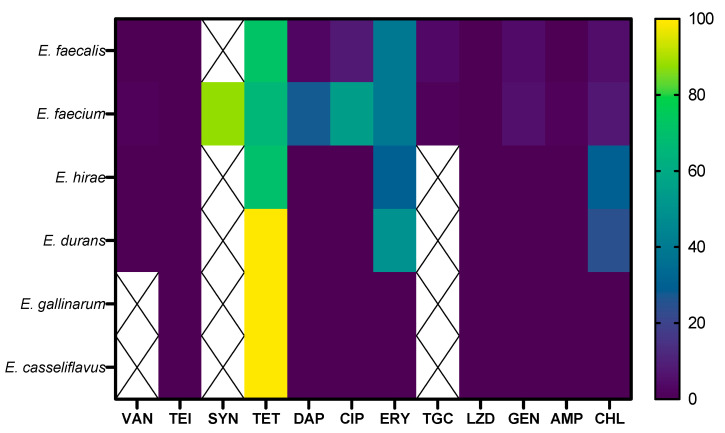
Heat map showing the frequency of antimicrobial resistance patterns across different *Enterococcus* species. VAN: vancomycin, TEI: teicoplanin, SYN: quinupristin–dalfopristin, TET: tetracycline, DAP: daptomycin, CIP: ciprofloxacin, ERY: erythromycin, TGC: tigecycline, LZD: linezolid, GEN: gentamicin, AMP: ampicillin, CHL: chloramphenicol. The rows represent different *Enterococcus* species, while the columns represent the antibiotics tested. The color gradient ranges from dark blue, indicating the lowest percentage of resistance, to yellow, indicating the highest percentage, with intermediate shades of blue–green representing increasing resistance levels. Cross-marked boxes indicate the absence of applicable breakpoints for specific species-antibiotic combinations.

Regarding resistance per species, statistically significant differences in MDR were shown (*p*-value < 0.05). The highest prevalence was in *E. faecium* (60.94%, 39/64), followed by *E. durans* (25%, 1/4), *E. faecalis* (12.69%, 17/134), and *E. hirae* (10%, 1/10). No MDR was observed in *E. gallinarum* (0/2) or *E. casseliflavus* (0/1). Varying levels of antibiotic resistance were also noted across species. As shown in Figure 2, *E. faecalis* exhibited high resistance frequencies (>50%) to TET (96/134). Similarly, *E. faecium* demonstrated high resistance to SYN (87.5%, 56/64) and significant resistance to CIP (35/64), DAP (18/64), and ERY (25/64). *E. hirae* showed resistance to ERY and CHL (33%, 3/10 for both) and high resistance to TET (70%). In *E. durans*, 100% resistance to TET (4/4) and 50% resistance to ERY (2/4) were observed. Isolates of *E. gallinarum* and *E. casseliflavus* showed 100% resistance to TET (2/2 and 1/1, respectively).

Statistically significant differences were observed in the resistance to each antibiotic across the *Enterococcus* species (*p*-value < 0.05) (Table 3). For VAN, almost no resistance was detected in any species, with only one sample isolated from *E. faecium* showing phenotypic resistance. Similarly, no resistance to TEI was observed in any species. High levels of resistance to SYN were noted in *E. faecium* (87.5%), while data were unavailable (NA) for other species. Regarding TET, resistance frequencies were highest in *E. durans*, *E. gallinarum*, and *E. casseliflavus* (100%) compared to *E. faecalis* (71.64%), *E. hirae* (70%), and *E. faecium* (65.63%), with significant differences among these groups. For DAP, *E. faecium* exhibited significantly higher resistance (28.13%) than other species. CIP resistance was significantly higher in *E. faecium* (54.69%) compared to *E. faecalis* (8.21%). In the case of ERY, moderate resistance was observed in most species, including *E. faecalis* (39.55%), *E. faecium* (39.06%), *E. hirae* (30%), and *E. durans* (50%), with significant differences compared to *E. gallinarum* and *E. casseliflavus*, where no resistance was observed.

Minimal resistance to TGC was observed in *E. faecalis* (3.73%) and *E. faecium* (1.56%), while data were unavailable (NA) for other species. No resistance to LZD was detected in any species. For GEN, low resistance rates were noted in *E. faecalis* (4.48%) and *E. faecium* (6.25%). AMP resistance was minimal, with only 1.56% of *E. faecium* strains showing resistance. Resistance to CHL was highest in *E. hirae* (30%) and *E. durans* (25%), followed by *E. faecalis* (5.22%) and *E. faecium* (7.81%), with statistically significant differences among species.

**Figure 3:**
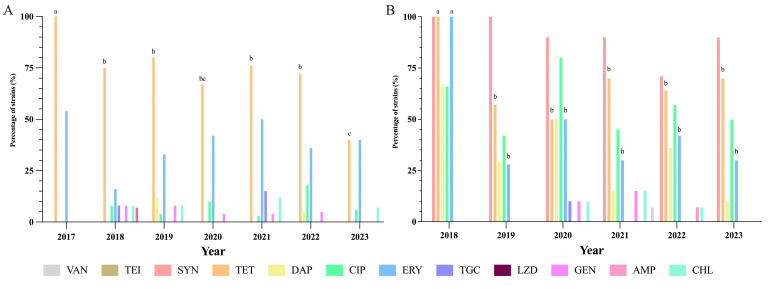
Evolution of antimicrobial resistance in *E. faecalis* (**A**) and *E. faecium* (**B**) over a six- or seven-year period (2017/8–2023). ^abc^: Superscripts show statistically significant differences in resistances in *Enterococcus* species within antibiotics per each year (*p* < 0.001). VAN: vancomycin, TEI: teicoplanin, SYN: quinupristin–dalfopristin (Only for *E. faecium* isolates), TET: tetracycline, DAP: daptomycin, CIP: ciprofloxacin, ERY: erythromycin, TGC: tigecycline, LZD: linezolid, GEN: gentamicin, AMP: ampicillin, CHL: chloramphenicol.

Regarding the evolution of antimicrobial resistances by year and species, Figure 3 illustrates the trends observed in both *E. faecalis* and *E. faecium*. For other species, analyzing this trend was challenging due to limited sample sizes and the lack of epidemiological breakpoints. However, in the case of *E. gallinarum*, despite having only two strains (isolated in 2019 and 2023), both showed resistance to the same antibiotics, indicating potential stability in resistance patterns over time.

For *E. faecalis*, resistance to TET decreased from 100% in 2017 to 40% in 2023. TGC resistance rose to 15.38% in 2021 but dropped to 0% in 2023. ERY resistance fluctuated from 57.14% in 2017 to 40% in 2023. CIP resistance peaked at 18.18% in 2022, then decreased to 6.67% in 2023. DAP resistance was detected only in 2019. GEN resistance remained low, while CHL resistance peaked at 11.54% in 2021, dropping to 6.67% in 2023. No SYN resistance data were available for *E. faecalis*.

Regarding *E. faecium*, TET resistance decreased from 100% in 2018 to 70% in 2023. ERY resistance decreased from 100% to 30%. CIP resistance slightly decreased from 66.67% to 50%. SYN resistance remained high, and DAP resistance fell from 66.67% in 2018 to 10% in 2023. VAN resistance appeared in 2022 (7.14%) but not in 2023. GEN and CHL resistance peaked at 15% in 2021, dropping to 0% in 2023.

A total of 66 *Enterococcus* isolates (26 from *E. faecalis*, 27 from *E. faecium*, 2 from *E. gallinarum*, and 1 from *E. casseliflavus*) which showed MIC values above or near the cohort threshold for phenotypic resistance to VAN were selected for the genotypic analysis of vancomycin resistance genes (*vanA*, *vanB*, *vanC*, and *vanD*). Among these, four *Enterococcus* strains (1.9%) turned out to be positive to the *vanB* gene (two from *E. gallinarum*, one from *E. faecium*, and one from *E. casseliflavus*) as the positive control, and the same two *vanB*-positive *E. gallinarum* strains also were positive to *vanC*. However, all the strains were negative to both the *vanA* and *vanD* genes. In comparison to the phenotypic results, we found that only one *E. faecium* strain evidenced phenotypic resistance to vancomycin according to the cohorts published by CLSI in 2024, which coincides with the strain positive to *vanB* gene. Nevertheless, two *E. gallinarum* and one *E. casseliflavus* strains that were shown to harbor the *vanB* gene demonstrated a low phenotypic expression that did not surpass the MIC currently reported for being considered resistant to VAN. Therefore, our results reveal an accordance between phenotypic and genotypic results and, fortunately, an expected, a very low number of strains resistant to VAN.

**Figure 4:**
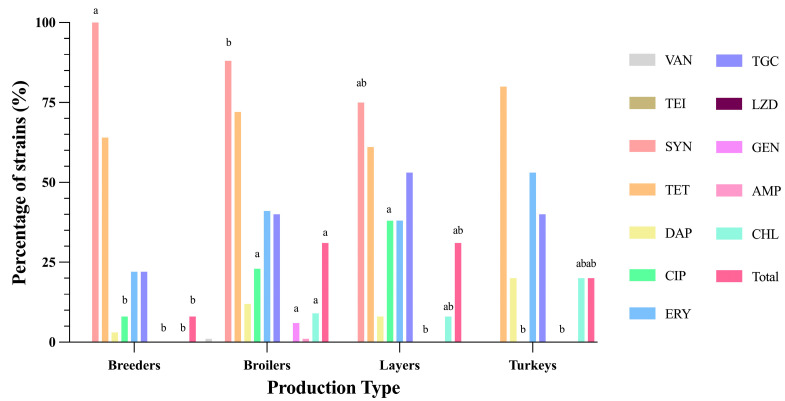
Levels of resistance per each production type. ^ab^: Superscripts show statistically significant differences in antibiotic resistance among poultry production type per each year (*p*-value < 0.001). VAN: vancomycin, TEI: teicoplanin, SYN: quinupristin–dalfopristin (only for *E. faecium* isolates), TET: tetracycline, DAP: daptomycin, CIP: ciprofloxacin, ERY: erythromycin, TGC: tigecycline (only for *E. faecalis* and *E. faecium* isolates), LZD: linezolid, GEN: gentamicin, AMP: ampicillin, CHL: chloramphenicol.

Concerning multidrug resistance (MDR) across production types, statistically significant differences (*p*-value < 0.05) were observed. Broilers exhibited the highest percentage of MDR strains (31.06%), closely followed by layers (30.77%). Turkeys demonstrated moderate MDR levels at 20%, while breeders displayed the lowest MDR percentage (8.33%). Levels of resistance per each production type are shown in Figure 4. Breeders exhibited 100% SYN resistance (specific to *E. faecium* isolates) but generally lower resistance to other antibiotics. Broilers demonstrated a broader resistance profile, with significant levels for TET (72.67%). Layers were particularly resistant to TET (61.54%), ERY (53.85%), and CIP (38.46%). Turkeys demonstrated the highest resistance to TET (80%), DAP (20%), and CHL (20%). Overall, broilers and turkeys exhibit broader and higher resistance patterns compared to breeders and layers. Turkeys, in particular, showed resistance to fewer antibiotics but with higher rates. Broilers and layers, in contrast, displayed more diverse resistance profiles.

## 3. Discussion

The present study assessed the relative prevalence, the distribution of AMR profiles and the virulence characteristics of *Enterococcus* strains isolated from various poultry production systems over a seven-year period (2017–2023). The results of this study show an increase in the incidence of *Enterococcus* isolates over the years, with *E. faecalis* (62.3%) and *E. faecium* (29.77%) being the predominant species. Smaller proportions of other species, including *E. hirae*, *E. durans*, *E. gallinarum*, and *E. casseliflavus*, were also detected. These findings align with those reported by Wigmore et al., who observed a relative prevalence of 42.8% and 32.28% for *E. faecalis* and *E. faecium*, respectively, in Australia [38]. Similarly, in Portugal, *E. faecalis* showed a relative prevalence of 51%, while *E. faecium* showed a prevalence of 44% (n = 76/174) [39]. Velhner et al. observed a relative prevalence of 37.5% for *E. faecalis* and 42.5% for *E. faecium* in poultry farms from Serbia [40]. While the prevalences of these species vary across regions, a consistent trend of increasing prevalence in poultry farming has been observed worldwide [6,14,23]. This regional variability highlights the influence of local factors on the distribution of *Enterococcus* species and underscores the importance of localized surveillance and targeted interventions to better understand and address these trends.

In terms of poultry production systems, 74.88% of the isolated strains were obtained from broilers, from which 31.16% were from one-week-old chicks. This observation is consistent with the findings of Stępień-Pyśniak et al., who reported enterococci detection in 88.1% of broilers, with the highest isolation frequency occurring in birds aged from 1 to 3 days [23]. Their study also identified *E. faecalis* as the most isolated species. Concerning breeders, 88.9% of their isolates were also identified as *E. faecalis*, suggesting them as a significant reservoir and source of transmission of antimicrobial-resistant pathogens throughout the poultry industry [41]. Among the less common *Enterococcus* species (*E. hirae*, *E. durans*, *E. gallinarum*, and *E. casseliflavus*), the low detection rates align with the findings from other studies. For example, Rehman et al. and Prakash et al. reported a low incidence of these strains in broiler chickens and humans, respectively [30,42]. These strains are often associated with serious conditions, such as bacteremia, encephalomalacia, necrosis, osteomyelitis, and endocarditis [14,23].

The results of the mannitol fermentation tests for *Enterococcus* species isolated from poultry revealed a high metabolic capacity, particularly in *E. faecalis* (99.25%) and *E. faecium* (96.88%). Quiloan et al. highlighted the ability of enterococci to grow in MSA due to their salt tolerance [43]. However, contrary to their findings, our results obtained showed that *E. faecium* isolates were also capable of fermenting mannitol. This metabolic ability may be associated with their pathogenic potential and adaptability to diverse environments. In this context, Nocera et al. isolated and differentiated pathogenic staphylococci and enterococci strains using MSA [44], demonstrating the relationship between the potential virulence of *Enterococcus* strains and their ability to degrade mannitol. In addition, Olsen et al. and Semedo-Lemsaddek et al. highlighted the role of poultry-derived *E. faecalis* with the ability to grow in MSA as a reservoir of virulence-associated genes, such as *gelE*, which may be transferred to human isolates [45,46]. Similarly, Silva et al. and Hasan et al. emphasized the food safety risks and public health implications of *Enterococcus* species from poultry environments, which harbor virulence factors and emerging MDR lineages [47,48].

Analysis of MDR phenotypic patterns revealed a diverse range of resistance profiles, with 26.98% of isolates classified as MDR strains. Similar results were reported in Portugal (23.5%) and Serbia (35%) [40,46]. In contrast, higher MDR prevalence was documented in other studies. Mudenda et al. reported an 86% prevalence of MDR *Enterococcus* isolates from layer hens in Zambia, while Stępień-Pyśniak et al. found even higher MDR rates, with 82.9% in Dutch poultry and 74.3% in Polish poultry [49,50].

Among the most prevalent resistances, *E. faecium* isolates showed an 87.5% resistance rate to SYN. This finding aligns with reports indicating that SYN, a streptogramin antibiotic, is only effective against *E. faecium*, as *E. faecalis* is intrinsically resistant due to the presence of the *lsa* gene [51,52]. This intrinsic resistance explains the absence of epidemiological breakpoints for E. faecalis isolates.

In relation to TET resistance, all *Enterococcus* species from this study showed high levels of resistance, ranging from 70% to 100%. Similar results were reported in chicken meat in Spain, which showed a 70.27% of *Enterococcus* spp. resistance against TET [53]. A similar percentage was found by Noh et al. in isolates from broiler breeder farms [42]. Additionally, Rebelo et al. reported that in 2018, *Enterococcus* isolates exhibited approximately 90% resistance to TET, coinciding with the years of the resistance’s peak detected for this antibiotic in this current study and with the start of data collection [39]. This resistance is attributed to the frequent use of this antimicrobial in farm animals for therapeutic purposes. The latest report from the European Medicines Agency (EMA) showed that TET together with penicillin were the most used antimicrobials, accounting for 52.7 and 34.7 mg/PCU (population correction unit), respectively [54]. Interestingly, a significant reduction in TET resistance was shown in this period for *E. faecalis* (from 100% to 70% (2017–2023) and *E. faecium* (from 100% to 40% (2018–2023).

Although primarily studied in human medicine, DAP resistance has also been reported in animal farming contexts. Gião et al. identified a resistance rate of 0.68% (n = 2) in *E. faecalis* strains from animal farming [55]. In poultry production, Diarra et al. reported a resistance rate of 2.90% (n = 2) in broiler production, including one strain each of either *E. faecium* or *E. faecalis*. Notably, the data presented in this investigation show a significantly higher resistance rate of 10.23% (n = 22) among the total strains analyzed, comprising 18 *E. faecium* and 4 *E. faecalis* strains [56]. DAP, an antibiotic without approved veterinary formulations in the European Union, has not shown cross-resistance with veterinary-approved antibiotic classes. Despite this fact, the mechanisms responsible for reducing daptomycin susceptibility in enterococci are not yet fully understood. The evidence links non-susceptibility to mutations in multiple genes [55]. In addition, laboratory studies by Prater et al. and Zeng et al. have further demonstrated that antimicrobial resistance can emerge following exposure to the antibiotic [57,58]. These findings highlight the need for continued research into the emergence and mechanisms of DAP resistance, particularly given its implications for both human and veterinary medicine.

With respect to TGC resistance, varying trends were observed across different countries and timeframes. In this study, a resistance rate of 3.03% was observed in *Enterococcus* isolates from Spanish poultry, with *E. faecalis* being the predominant species, accounting for five out of the six resistant isolates detected. Comparing these results to other published studies, Poland reported a 0% resistance rate [59]. However, de Jong et al. documented higher resistance levels in chickens, with an increase from 7.2% in 2008–2009 to 11.4% in 2013–2014 [60]. These findings highlight significant regional and temporal variations in TGC resistance.

For VAN resistance, a single strain of *E. faecium* was identified, representing 0.47% of the total isolates analyzed. According to EUCAST’s 2023 antimicrobial susceptibility breakpoints, *E. casseliflavus* and *E. gallinarum* would also be classified as resistant due to their MIC values of ≥4 mg/L [61]. However, the EUCAST guidelines from 2024 have excluded these species, recognizing their intrinsic resistance to VAN, as previously reported by Courvalin and Rehman et al. [30,62,63]. In our analysis, *E. gallinarum* exhibited 100% resistance, consistent with the already intrinsic resistance [64]. This distinction underscores the importance of recognizing intrinsic resistance mechanisms in these species for the accurate interpretation of clinical results.

In addition to the phenotypic analysis, we analyzed the expression of the *vanA*, *B*, *C*, and *D* genes, because they are the most common AMR genes to VAN that have been analyzed and found in the literature for clinical cases, and because VAN is a last-resort antibiotic used for human health. In this sense, the ATCC VRE panel (ATCC MP-1) is composed of 15 *Enterococcus* strains representing only the *vanA, vanB*, and *vanC* genotypes.

Genotypic analysis evidenced the presence of both *vanB* and *vanC* vancomycin-resistant genes in *E. gallinarum* isolates, while *vanB* in only one isolate from *E. casseliflavus* and *E. faecium*. In comparison to the low percentages found in our study, Heidari et al. studied the detection of *vanA* and *vanB* genes in *Enterococcal* strains isolated from burn patients without finding any of them [65], whereas Lata et al. reported a 27% frequency of the *vanB* gene in resistant *E. faecalis* isolates from an Indian river running along a north Indian city landscape, which was a disturbing and alarming score for human health. In comparison, no *E. faecalis* strain harboring the *vanB* gene was evidenced in this study [66]. In addition, Furuya et al. reported 2 *E. faecium* isolates (3.3%) from 60 clinical cases harboring the *vanB* gene, with 1 documented as an imported case, and the other as a nosocomial case, whereas a 96.7% frequency of *E. faecium* isolates harbored the *vanA* gene, which suggested that this gene was more related to VRE outbreaks in humans [67]. Moreover, Shaker et al. analyzed diarrheic pet animals’ samples to detect VRE both phenotypically and genotypically, finding that 11.8% of samples were positive for VRE and detecting the *vanB* gene in 4 out 10 *E. faecalis* strains with a prevalence rate of 40% [68]. In our study, one *E. faecium* strain was detected harboring the *vanB* gene, but none of the *E. faecalis* strains were positive to *van* genes.

Furthermore, Yean et al., reported that *vanA*-type strains are resistant to high levels of both VAN and TEI antimicrobials (MIC ≥ 64 µg/mL and >16 µg/mL, respectively), *vanB*-type strains are resistant to a wide range of VAN concentration (MIC between 4 to ≥1024 µg/mL) and are susceptible to TEI, and *vanD*-type strains are resistant to moderate levels of VAN (MIC 128 µg/mL) and susceptible to TEI, while *vanC*, *vanE*, and *vanG*-type strains exhibit low-level resistance to VAN [69]. In the same context, Ben Yahia et al. analyzed acquired VAN resistance-encoding genes (*vanA*, *B*, *D*) and intrinsic VAN resistance-encoding genes (*vanC-1* and *vanC-2/3* linked to the species *E. gallinarum* and *E. casseliflavus*/*flavescens*, respectively) in wild birds, detecting only *E. faecalis* strains harboring *vanA* and *vanB* genes [70]. Therefore, these investigations support our decision to analyze the *vanA*, *B*, and *D* genes for acquired resistance to VAN in *Enterococcus* strains, and the intrinsic resistance gene *vanC*, found in strains with low-level resistance to VAN, as well as our results regarding the detection of the *vanC* gene in *E. gallinarum* strains.

Concerning TGC, DAP, VAN, and LIN, these antibiotics are classified under Category A in the EMA’s, which includes antimicrobials not approved for veterinary use. This categorization likely reflects indirect selection pressures rather than direct usage in animal farming. Despite strict regulations enforced under the EMA’s Antimicrobial Advice Ad Hoc Expert Group (AMEG) guidelines, the detection of resistance to DAP and TGC underscores the need for vigilant monitoring and control programs. Such initiatives are essential to address indirect drivers of resistance in poultry farming and to safeguard these critical antimicrobials for human health [71].

In terms of production types, Stępień-Pyśniak et al. reported the following distribution of *Enterococcus* isolates in Poland: broilers (88.1%), laying hens (5.3%), turkeys (3.9%), breeding hens (2.2%), and geese (0.4%) [23]. This is in line with our results, showing that 74.88% of *Enterococcus* isolates came from broilers, 2.33% from turkeys, and 6.05% from layers. However, a notable difference was observed in breeders, where the detection rate in this study was 16.74%, compared to the much lower percentage reported. The high prevalence of *Enterococcus* species in broilers is further supported by other investigations. For example, Velhner et al. reported a 60% prevalence in broilers from Serbia, reflecting a general trend of higher *Enterococcus* isolation rates in this production type [40].

In summary, this study encompasses *Enterococcus* isolates collected from poultry between 2017 and 2023, with resistance patterns assessed using the most recent guidelines (2024). During this period, certain antibiotic resistance breakpoints have changed. For TGC, the CLSI breakpoint was adjusted in 2019 from ≤0.5 mg/L to ≤0.25 mg/L, while for SYN the EUCAST breakpoint was reduced in 2019 from ≤4 mg/L to ≤1 mg/L. For DAP, the CLSI introduced a resistance cutoff of ≥8 mg/L in 2019. To ensure consistency and rigor, we applied the most restrictive breakpoints retroactively, providing a standardized and conservative analysis of resistance patterns.

On the other hand, surveillance of MDR *Enterococcus* isolates in poultry production is crucial for mitigating antimicrobial resistance and safeguarding public health. Nonetheless, in 2022, the EFSA reported the lack of formal surveillance programs for *E. faecalis* in poultry, with diagnostic resistance data rarely published [72]. The transmission of resistances from *Enterococcus* isolates from poultry to humans can be a significant public health concern. These bacteria, particularly *E. faecalis* and *E. faecium*, can be transmitted through direct contact, contaminated food, and environmental exposure. In this regard, Aun et al. describes that poultry strains of *E. faecalis* are closely related and harbor mobile antibiotic resistance genes, which contribute to the spread of resistance across different reservoirs [73]. Their study highlights a significant genetic exchange and underscores the potential for transmitting antimicrobial resistance genes from poultry to humans. In the same context, Ben Yahia et al. revealed a connection between the underlying genetic mobile elements (such as AMR genes and specifically van genes) that confer antibiotic resistance and an increased virulence found in *E. faecalis* strains, also detected by the presence of certain genes, such as *gelE*, *hyl*, *ace* and *esp*. They stated that this connection contributes to the spread of MDR bacteria in the environment [70]. In addition, Ben Said et al. documented AMR in enterococci of food-producing animals and water samples in Tunisia, suggesting a source of transmission of resistant *Enterococcus* strains from animals to humans [74]. These findings might pose a risk to human health considering that some species are responsible for nosocomial infections [75].

Although South Korea has implemented an antimicrobial resistance monitoring program since 2003, gaps remain in assessing resistance at critical stages, such as in the broiler parent stock [41]. Thus, all these observations regarding rising resistance levels highlight the critical need for comprehensive and standardized surveillance programs to track the prevalence and antimicrobial resistance patterns of *Enterococcus* species in poultry production systems globally, as well as a continuous monitoring of both clinical and agricultural settings to preserve the effectiveness of antimicrobials, prevent further restrictions on their use [76], and ensure the effective management of this growing public health concern.

Finally, discrepancies among findings from different countries and studies regarding AMR in *Enterococcus* species may stem from the absence of a standardized database or incomplete data for certain antibiotics.

These differences are expected in the case of certain species, such as *E. hirae* and *E. durans*, which are predominantly associated with the poultry industry rather than humans, given that the antibiotics used in this study are primarily intended for human medicine. Nevertheless, resistance is not solely a strain-specific issue but involves the potential transfer of resistance genes to the broader microbiota or concomitant bacteria.

Regional variability in AMR profiles and resistance mechanisms underscores the influence of localized factors, including environment reservoirs, farming practices, antimicrobial usage, and biosecurity measures. The results from this study showed the percentage of samples from specific locations in Spain. To better compare the prevalence of AMR across the entire country, greater representation from geographic locations, such as Catalonia and Aragón, which are also significant in Spanish poultry farming, would enhance the scope of the study. Due to the lack of knowledge in differentiating pathogenic strains from commensal ones, other techniques, such as molecular biology or sequencing, would be highly useful to clarify this issue. Further studies are necessary to establish the relationship between virulent and/or resistant strains, which would provide improved insights into the prevalence of VAGs and virulent strains, ultimately helping to inform national control measures.

The results of the study emphasize the importance of targeted interventions, stricter regulations, and enhanced AMR surveillance to guide antibiotic stewardship and mitigate resistance risks. First, standardized global surveillance programs must be established to track AMR trends and resistance mechanisms comprehensively. Second, promoting the responsible use of antimicrobials in poultry production is essential to minimize selection pressures and reduce resistance development. Third, adopting a One Health perspective that integrates human, animal, and environmental health data will facilitate a holistic understanding of resistance dynamics and enable coordinated action across sectors. Finally, investing in research to develop alternative strategies for infection prevention and control, such as vaccines, probiotics, and improved biosecurity measures, could reduce our reliance on antimicrobials in poultry farming. These steps are vital for mitigating the spread of resistance, preserving the efficacy of existing antimicrobials, and safeguarding both animal and human health. Addressing these challenges with urgency and collaboration is essential to curbing the global threat of antimicrobial resistance.

## 4. Materials and Methods

### 4.1. Bacteria Selection

In this study, 215 *Enterococcus* strains, isolated from clinical cases from Spanish poultry farms between 2017 and 2023, were selected. The farms selected were from conventional production systems. Of the total samples, 41% came from the Valencian Community, 21% came from Andalusia, 18% came from Castilla-La Mancha, 8% came from Murcia, 4% came from Castilla y León, 3% came from Catalonia, and 1% came from Aragón. The production types included broilers (n = 161), turkeys (n = 5), breeders (n = 36), and layers (n = 13), with age categories ranging from 1 to 6 weeks and beyond for broilers and turkeys while breeders and layers were categorized into rearing (1–24 weeks) and production (25–60 or 25–100 weeks) periods (Table 4). The relative prevalence was defined as the relative number of *Enterococcus* species within the total isolates of *Enterococcus*.

### 4.2. Bacteria and Culture Identification

The strains were recovered from the bank repository of the CECAV strain bank. Cryopreserved strains were reactivated in brain–heart infusion (BHI) agar and incubated for 24 h at 37 °C. Afterwards, the cultures were plated onto Columbia blood sheep agar (CA) (Oxoid Deutschland GmbH, Wesel, Germany) and incubated at 37 °C to isolate single colonies. Single colonies were used for further characterization analysis. Then, the identification of *Enterococcus* isolates was performed using matrix-assisted laser desorption ionization time-of-flight (MALDI-TOF) analysis.

### 4.3. Mannitol Fermentation Assesment

**Figure 5:**
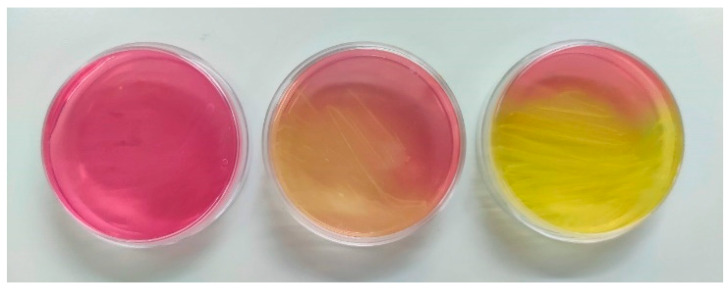
From left to right: red medium with pale pink colonies indicating no mannitol fermentation (-); orange-yellowish medium showing partial mannitol fermentation (+); bright yellow medium demonstrating complete mannitol fermentation (++).

Mannitol fermentation capacity was evaluated by culturing *Enterococcus* isolates in a mannitol salt agar (MSA) selective and differential medium containing phenol red as a pH indicator, following the protocol described by Quiloan et al. [43]. Mannitol fermentation turns the medium yellow due to acid production, allowing differentiation between fermenting and non-fermenting bacterial isolates, Figure 5. To this end, isolates were streaked onto MSA plates and incubated at 37 °C for 24–48 h. After incubation, bacterial growth and colour changes were observed. A red-to-yellow shift indicated mannitol fermentation and acid production.

### 4.4. Antimicrobial Susceptibility Test

The antimicrobial susceptibility test was conducted using a Sensititre™ ^®^ EU Surveillance *Enterococcus* EUVENC Plate (Thermofisher Scientific, West Sussex, UK). Briefly, 3–5 colonies were picked from the bacterial culture and suspended in sterile water to achieve a 0.5 McFarland standard measure using a nephelometer. A 10 μL aliquot of this suspension was then mixed into 11mL of Mueller–Hinton broth (MHB). The inoculum was then dispensed onto the Sensititre plate using a multi-channel pipette, with 100 μL volume per well. The Sensititre plate was then sealed and incubated at 34–36 °C for 24 h to ensure the detection of vancomycin-resistant *Enterococcus* spp. The microdilution plate contained the following antimicrobials: VAN (1–128 µg/mL), TEI (0.5–64 µg/mL), SYN (0.5–64 µg/mL), TET (1–128 µg/mL), DAP (0.25–32 µg/mL), CIP (0.12–16 µg/mL), ERY (1–128 µg/mL), TGC (0.03–4 µg/mL), LZD (0.5–64 µg/mL), GEN (8–1024 µg/mL), AMP (0.5–64 µg/mL), and CHL (4–128 µg/mL). For the analysis of the results, breakpoints were considered following the recommendations of EUCAST from 2024 [62]. In cases where specific breakpoints were not reflected in the EUCAST guidelines, CLSI M100-ED34:2024 was used as a reference [77] (Table 5). An isolate was defined as MDR if it showed resistance to at least three or more antimicrobial families of antibiotics [78].

### 4.5. Detection of Vancomycin-Resistant Genes with RT-PCR

To detect VAN resistance genes in *Enterococcus* species, bacterial cultures were grown in LB broth at 37 °C and 120 rpm for four hours. DNA extraction was performed using the MagMax CORE kit nucleic acid purification (Applied Biosystems, Austin, TX, USA) following the manufacturer’s instructions, requiring 200 µL of bacterial culture and 90 µL of elution buffer per sample. After that, these samples were analyzed using real-time PCR (RT-PCR).

Sequences of four resistance genes to VAN (*vanA*, *vanB*, *vanC*, and *vanD*) were looked for using specific primers for the amplification of these genes, as documented in Table 6. RT-PCR assays were developed in four single reactions for each *Enterococcus* strain, and each reaction was carried out in a total volume of 10 µL containing 2 µL of template DNA, 0.2 µM of each forward and reverse primer, 5 µL SYBR GreenER qPCR SuperMix Universal (Invitrogen, Life technologies, Carlsbad, CA, USA), and 37.5 nM ROX reference dye, with the rest of volume filled with nuclease-free water.

RT-PCR analyses were performed using the QuantStudio 5 equipment (Applied Biosystems, Foster City, CA, USA) with the following cycling conditions: 50 °C for 2 min for uracil DNA glycosylase (UDG) incubation, 95 °C for 10 min for UDG inactivation and DNA polymerase activation, and 40 cycles of 2 steps (denaturation at 95 °C for 15 s, followed by DNA polymerization at 60 °C for 1 min). A cycle threshold (Ct) of more than 32 was considered as a negative gene detection for each sample according to the limit reported by Pholwat et al. for antibiotic resistance genes [79]. The *E. faecalis* strain CECT 8120 was acquired and used as a positive control for the *vanB* gene, whereas for the rest of the genes without positive controls, presumptive positives to either *vanA*, *vanC*, or *vanD* were confirmed by conventional PCR and electrophoresis on 1% agarose gel.

**Table 6:** Primers used in the RT-PCR for the detection of vancomycin-resistance genes in *Enterococcus* spp. strains.

Gene	Primer (5′ to 3′)	Product Size (bp)	GenBank Accession Number	Reference
*vanA*	F-AATTGCTATTCAGCTGTACTC	684	JN029954.1, EF206284.1, X56895.1	Poeta et al. [80]
	R-ACGGGCTAGACCTCTACAGCC			
*vanB*	F-AATGCGGGGAGGATGGTGCG	428	U00456.1, KF823968.1, AF192329.1	Yean et al. [69]
	R-GATGCGGAAGATACCGTGGC			
*vanC*	F-GCTGCCTCCGCATTATGTATGAA	349	AF162694.1, KU296945.1	Yean et al. [69]
	R-GAGAAATCGCATCACAAGCA			
*vanD*	F-CGTATGTGGGATGCGATATTCAA	234	AF130997.1, AF175293.1	Yean et al. [69]
	R-CTTCGATTGCTGCCTGCAGTT			

### 4.6. Statistical Analysis

AMR patterns were analyzed using a generalized linear model (GLM) to evaluate temporal trends in AMR rates from 2017 to 2023 within each poultry production type (broilers, turkeys, layers, and breeders). The GLM also facilitated comparisons of AMR profiles across production types and for specific antibiotics within the same year. Statistical significance was defined as *p*-value ≤ 0.05. All analyses were performed using SPSS version 30.0 (SPSS Inc., Chicago, IL, USA). The alluvial diagram was created using RAWGraphs v.2, an open-source data visualization platform [37].

## 5. Conclusions

The findings reveal significant AMR, with a notable proportion of MDR strains and resistance to critical antibiotics, including tetracycline, daptomycin, and tigecycline.

These trends underline the critical role of poultry, especially broilers and breeders, as reservoirs and potential transmission sources of resistant pathogens to humans and the environment. The detection of vancomycin-resistant genes (*vanB* and *vanC*) and resistance to antibiotics not approved for veterinary use raises serious public health concerns, emphasizing the risk of cross-species transmission of resistance and its implications for human medicine. To combat these challenges effectively, several critical actions are needed.

## Figures and Tables

**Table 1:** Distribution of *Enterococcus* isolates during 2017–2023 (n and percentage %).

Year	*E. faecalis*	*E. faecium*	*E. hirae*	*E. durans*	*E. gallinarum*	*E. casseliflavus*	Total
2017	7 (100%)	-	-	-	-	-	7
2018	12 (80.00%)	3 (20.00%)	-	-	-	-	15
2019	24 (72.73%)	7 (21.21%)	1 (3.03%)	-	1 (3.03%)	-	33
2020	28 (71.79%)	10 (25.64%)	-	-	-	1 (2.56%)	39
2021	26 (50.98%)	20 (39.22%)	3 (5.88%)	2 (3.92%)	-	-	51
2022	22 (56.41%)	14 (35.90%)	2 (5.13%)	1 (2.56%)	-	-	39
2023	15 (48.39%)	10 (32.26%)	4 (12.90%)	1 (3.23%)	1 (3.23%)	-	31
Total	134 (62.33%)	64 (29.77%)	10 (4.65%)	4 (1.86%)	2 (0.93%)	1 (0.47%)	215

**Table 2:** Results of *Enterococcus* MSA fermentation.

Specie	n	Results					
		++		+		-	
		n	%	n	%	n	%
*E. faecalis*	134	133	99.25	1	0.75	0	0
*E. faecium*	64	62	96.88	1	1.56	1	1.56
*E. hirae*	10	0	0	4	40	6	60
*E. durans*	4	0	0	2	50	2	50
*E. gallinarum*	2	2	100	0	0	0	0
*E. casseliflavus*	1	1	100	0	0	0	0

++ High mannitol fermentation capacity, + medium fermentation capacity, - unable to ferment mannitol.

**Table 3:** AMR trend by antibiotic and *Enterococcus* specie.

Antibiotic	*E. faecalis*	*E. faecium*	*E. hirae*	*E. durans*	*E. gallinarum*	*E. casseliflavus*
	(n = 134)	(n = 64)	(n = 10)	(n = 4)	(n = 2)	(n = 1)
	Resistance rates %					
VAN	0	1.56	0	0	0	0
TEI	0	0	0	0	0	0
SYN	NA	87.5	NA	NA	NA	NA
TET	71.64 ^b^	65.63 ^b^	70 ^b^	100 ^a^	100 ^a^	100 ^a^
DAP	2.99 ^b^	28.13 ^a^	0 ^c^	0 ^c^	0 ^c^	0 ^c^
CIP	8.21 ^b^	54.69 ^a^	0 ^c^	0 ^c^	0 ^c^	0 ^c^
ERY	39.55 ^a^	39.06 ^a^	30 ^a^	50 ^a^	0 ^b^	0 ^b^
TGC	3.73	1.56	NA	NA	NA	NA
LZD	0	0	0	0	0	0
GEN	4.48	6.25	0	0	0	0
AMP	0	1.56	0	0	0	0
CHL	5.22 ^a^	7.81 ^a^	30 ^a^	25 ^ab^	0 ^b^	0 ^b^

^abc^: Superscripts in each row show statistically significant differences per each antibiotic within *Enterococcus* species (*p*-value < 0.001). VAN: vancomycin, TEI: teicoplanin, SYN: quinupristin–dalfopristin, TET: tetracycline, DAP: daptomycin, CIP: ciprofloxacin, ERY: erythromycin, TGC: tigecycline, LZD: linezolid, GEN: gentamicin, AMP: ampicillin, CHL: chloramphenicol. NA: not applicable.

**Table 4:** Clinical strains recovered from 2017 to 2023.

Production Type	Age (Weeks)	Year															
		2017		2018		2019		2020		2021		2022		2023		Total	
		n	%	n	%	n	%	n	%	n	%	n	%	n	%	n	%
**Broiler** **(74.88%)**	**1**	-	-	2	13.33	14	42.42	9	23.08	13	25.49	15	45.5	14	45.16	67	31.16
	**2**	-	-	-	-	1	3.03	2	5.13	9	17.65	2	6.06	2	6.45	16	7.44
	**3**	-	-	-	-	4	12.12	5	12.82	7	13.73	4	12.12	7	22.58	27	12.56
	**4**	-	-	1	6.67	1	3.03	3	7.69	5	9.80	3	9.09	2	6.45	15	6.98
	**5**	-	-	1	6.67	3	9.09	1	2.56	4	7.84	3	9.09	1	3.23	13	6.05
	**6**	-	-	2	13.33	5	15.15	3	7.69	3	5.88	3	9.09	-	-	16	7.44
	**>6**	-	-	2	13.33	2	6.06	-	-	3	5.88	-	-	-	-	7	3.26
**Turkeys** **(2.33%)**	**2**	-	-	-	-	-	-	2	5.13	-	-	-	-	-	-	2	0.93
	**3**	-	-	-	-	-	-	-	-	1	1.96	-	-	-	-	1	0.47
	**5**	-	-	-	-	2	6.06	-	-	-	-	-	-	-	-	2	0.93
**Breeders** **(16.74%)**	**Rearing period** **(1–24 wks)**	7	100	6	40	1	3.03	11	28.21	3	5.88	1	3.03	3	9.68	32	14.88
	**Production period** **(25–60 wks)**	-	-	1	6.67	-	-	-	-	1	1.96	2	6.06	-	-	4	1.86
**Layers** **(6.05%)**	**Rearing period** **(1–24 wks)**	-	-	-	-	-	-	2	5.13	-	-	3	9.09	1	3.23	6	2.79
	**Production period** **(25–60 wks)**	-	-	-	-	-	-	1	2.56	2	3.92	3	9.09	1	3.23	7	3.26
**Total**		7		15		33		39		51		33		31		215	

**Table 5:** Breakpoints used for the detection of AMR.

Antibiotic	Breakpoints (MIC) (µg/mL)					
	*E. faecium*	*E. faecalis*	*E. gallinarum*	*E. hirae*	*E. casseliflavus*	*E. durans*
VAN	>4 *	>4 *	NA	>4 *	NA	>4 *
TEI	>4 *	>4 *	>4 *	>4 *	>4 *	>4 *
SYN	>1 *^;^ **	NA	NA	NA	NA	NA
TET	≥16 **	≥16 **	≥16 **	≥16 **	≥16 **	≥16 **
DAP	≥8 **	≥8 **	≥8 **	≥8 **	≥8 **	≥8 **
CIP	>4 *	>4 *	>4 *	>4 *	>4 *	>4 *
ERY	≥8 **	≥8 **	≥8 **	≥8 **	≥8 **	≥8 **
TCG	>0.25 *	>0.25 *	NA	NA	NA	NA
LZD	>4 *	>4 *	>4 *	>4 *	>4 *	>4 *
GEN	Used to screen for the presence of aminoglycoside-modifying enzymes Negative test ≤ 128; Positive test > 128					
AMP	>8 *	>8 *	>8 *	>8 *	>8 *	>8 *
CHL	≥32 **	≥32 **	≥32 **	≥32 **	≥32 **	≥32 **

VAN: vancomycin, TEI: teicoplanin, SYN: quinupristin–dalfopristin, TET: tetracycline, DAP: daptomycin, CIP: ciprofloxacin, ERY: erythromycin, TGC: tigecycline, LZD: linezolid, GEN: gentamicin, AMP: ampicillin, CHL: chloramphenicol. *: data obtained from EUCAST 2024; **: data obtained from CLSI 2024; NA: No breakpoint indicated for this species in EUCAST/CLSI database.

## Data Availability

The original contributions presented in this study are included in the article. Further inquiries can be directed to the corresponding author.
